# Causal relationship between primary biliary cholangitis on osteoporosis: A two-sample Mendelian randomization study

**DOI:** 10.1097/MD.0000000000043164

**Published:** 2025-07-04

**Authors:** Weinan Liu, Yanling Dai, Jian Liu, Jiazhong Lin, Shoubao Wang

**Affiliations:** a Department of Orthopedics, The Affiliated People’s Hospital of Fujian University of Traditional Chinese Medicine, Fuzhou, China; b School of Nursing, Fujian University of Traditional Chinese Medicine, Fuzhou, China; c Department of Orthopedics, Huai’an Hospital Affiliated to Yangzhou University (The Fifth People’s Hospital of Huai’an), Huai’an, China.

**Keywords:** causal inference, Mendelian randomization, OP, PBC

## Abstract

This present study aims to investigate the relationship between primary biliary cholangitis (PBC) and osteoporosis (OP) using a two-sample Mendelian randomization (MR) analysis. A two-sample MR study was conducted to explore the association between PBC and OP. The inverse variance weighted test was primarily used to estimate causality. Horizontal pleiotropy was assessed using both MR-PRESSO and MR-Egger regression techniques. Cochran Q test was applied to examine heterogeneity among single nucleotide polymorphisms (SNPs), and a sensitivity analysis was performed to evaluate the impact of each individual SNP on the MR analysis results. The two-sample MR analysis result showed a significant association between PBC and OP, with an inverse variance weighted odds ratio of 1.098 (95% confidence interval: 1.049–1.150,and a *P* = 5.41e-05). MR-Egger regression indicated no bias due to genetic pleiotropy (intercept = 0.007, SE = 0.022, *P* = .754). Cochran Q test revealed no significant heterogeneity (Q = 27.494, *P* = .236). Furthermore, leave-one-out analysis confirmed the robustness of our findings, as the results remained consistent even when individual SNPs were excluded. This study provides evidence supporting the notion that PBC may increase the risk of OP, enhancing our understanding of the association between PBC and OP.

## 1. Introduction

Osteoporosis (OP) is a systemic bone disease characterized by reduced bone mass and deterioration of the trabecular structure, leading to increased bone fragility and a high incidence of fractures.^[1]^ Key risk factors include advanced age, hormonal imbalances (such as diabetes, thyroid issues, and insufficient vitamin D), low estrogen levels after menopause, abnormalities in growth hormone and insulin-like growth factor-1, poor nutrition, obesity, and medications that negatively impact bone health.^[2]^ OP often remains undiagnosed until complications, such as fractures, arise, impacting patient safety and quality of life, with a prevalence of 30% to 40% in women and 10% to 20% in men over 50 years of age.^[3]^ In Europe and the United States, OP fractures represent a significant public health challenge, with incidence rates approaching 33% in the general population and escalating to approximately 50% among postmenopausal women, thereby imposing substantial healthcare economic pressures.^[4]^ Current treatment for OP largely depends on pharmacological interventions, including bisphosphonates, calcitonin, estrogens, and estrogen receptor agonists, which have limited effectiveness and may induce adverse effects.^[5]^ Consequently, it is essential to identify additional OP risk factors and broaden the range of treatment options. OP is a multifactorial disease, and understanding its risk factors can significantly enhance prevention and management strategies. For instance, a study on the prevalence and risk factors of OP in Chinese postmenopausal women awaiting total knee arthroplasty highlighted that age, body mass index, and knee deformities are critical indicators of OP risk.^[6]^ Furthermore, the exploration of blood factors as biomarkers during the COVID-19 pandemic has opened new avenues for identifying individuals at risk for OP, suggesting that these biomarkers could facilitate quicker screening and monitoring.^[7]^ Moreover, the coexistence of OP and coronary artery disease in the elderly underscores the importance of recognizing comorbid conditions that may exacerbate OP.^[8]^ This relationship emphasizes the need for comprehensive assessments that consider both bone health and cardiovascular risk factors. Additionally, the management of OP can be improved by integrating various treatment modalities, including bisphosphonates and lifestyle modifications, which have been shown to mitigate the risks associated with this condition.^[9]^ In summary, a multifaceted approach that includes identifying risk factors, utilizing biomarkers, and expanding treatment options is crucial for effective OP management.

Primary biliary cholangitis (PBC) is an autoimmune liver disease characterized by small and medium-sized bile duct inflammation. Autoimmune hepatitis, as the main type of autoimmune liver diseases, has significant differences in incidence and prevalence among different countries and regions.^[10]^ PBC has a complex etiology involving genetic predisposition, environmental factors, and immune responses. Familial clustering and genome-wide association studies (GWAS) indicate that certain genetic variants, particularly within the human leukocyte antigen region, are associated with increased susceptibility to PBC, underscoring a genetic predisposition. Environmental triggers, such as infectious agents and exposure to xenobiotics, may initiate or intensify immune-mediated damage to the bile ducts in PBC. One of the major complications of PBC is OP, with research indicating that the incidence of OP in PBC patients is approximately 3.3 times higher than in the general population, with rates ranging from 20% to 52%.^[11]^ The pathogenesis of OP in PBC is distinct from that of age-related primary OP. In PBC, OP is mainly caused by a reduction in bone formation, unlike the increased bone resorption typically seen in postmenopausal OP.^[12]^ This difference is significant because it affects both treatment approaches and the overall understanding of bone health in patients with PBC. Patients with PBC frequently suffer from considerable health issues related to OP, resulting in a high incidence of fractures that can greatly diminish their quality of life.^[13]^ The development of OP in individuals with PBC is influenced by various factors. One significant contributor is the malabsorption of vital nutrients, especially vitamin D and calcium, both of which play crucial roles in maintaining bone health.^[13]^

The use of medications such as corticosteroids in certain patients can worsen bone loss, underscoring the importance of careful management of OP in this group.^[12]^ Current treatment strategies for OP in PBC primarily utilize therapies designed for postmenopausal OP, including bisphosphonates. However, the effectiveness of these treatments specifically for OP related to PBC is still unclear, as clinical trials have produced inconsistent results.^[12]^ Consequently, gaining a deeper understanding of the unique pathophysiology of OP in PBC may be important for developing targeted treatments to improve patient outcomes.^[12]^

Mendelian randomization (MR) provides a method for investigating causal relationships between exposures and outcomes based on Mendel second law of inheritance.^[14]^ In MR, the random assignment of genetic variants from parents to offspring serves as instrumental variables (IVs) for exposure, helping to address issues such as reverse causality, confounding, and biases that can affect observational studies.^[15]^ To conduct MR effectively, 3 conditions must be met: (1) the genetic IV should have a strong association with the exposure; (2) the genetic IV should not be associated with any other factors that may influence the outcome; and (3) the genetic IV should affect the outcome solely through the exposure, without other biological pathways.^[16]^ The present study aims to investigate the relationship between PBC and OP using MR, providing new perspectives for future research and treatment approaches.

## 2. Methods and materials

### 2.1. Study design overview

**Figure 1.**
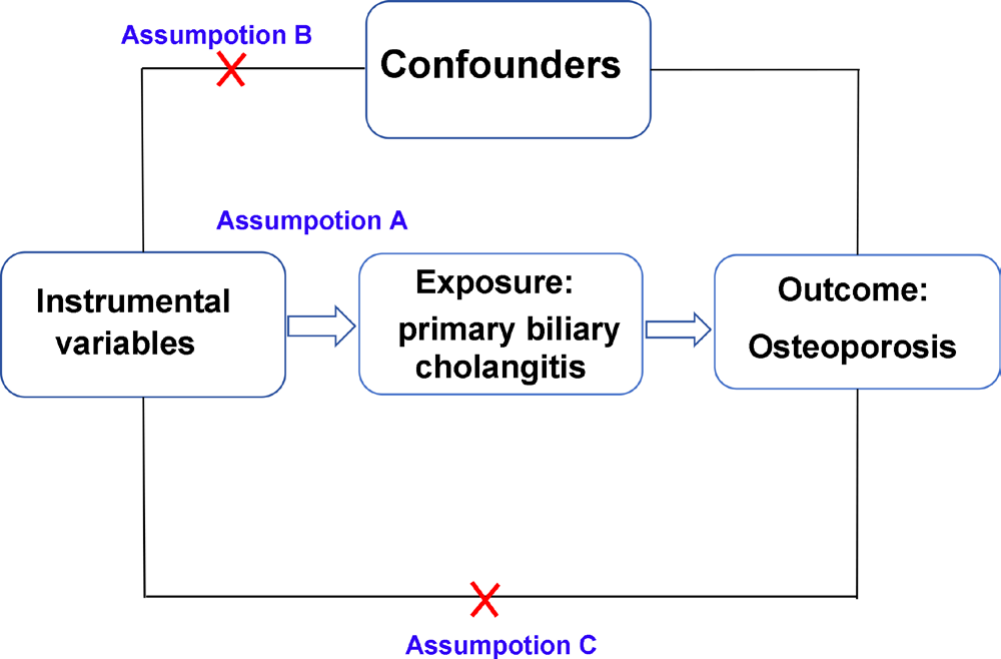
The study design examines the causal link between PBC and OP using MR, with 3 key assumptions. OP = osteoporosis, PBC = primary biliary cholangitis.

Data from approved, published studies were collected with informed consent from participants^[17]^; thus, no additional ethical approvals were required.^[18]^ This MR study analyzed the causal relationship between PBC and OP, using single nucleotide polymorphisms (SNPs) as IVs. Figure 1 illustrates the study design. By using SNPs as IVs, MR simulates the effects of randomized controlled trials to determine causal connections between the exposure (PBC) and the outcome (OP).

### 2.2. PBC and OP GWAS summary statistics

Information on PBC was obtained from the most recent and comprehensive GWAS meta-analysis, published in 2015 by HJ et al, which included 2764 cases and 10,475 controls.^[19]^ All the PBC data (ID: ebi-a-GCST003129) information were downloaded from the IEU Open GWAS project by using the “Two Sample MR” R package. The SNPS of the samples were retained must to meet the following criteria: (1) they were highly correlated with PBC at a genome-wide level (*P* < 5 × 10^-8^) and were not linked to each other to prevent bias from linkage disequilibrium (*r*² < 0.001 within a 10,000 kb window); (2) the F-statistic needs >10 to ensure reliability. Similarly, the OP data was also sourced from the IEU Open GWAS project, specifically from the ID: finn-b-M13_OP, which included 3203 cases and 209,575 controls. Detailed information on the exposure and outcome variables is provided in Table 1.

**Table 1:** The information from the GWAS dataset.

Phenotype	Consortium	Year	Case(N)	Sample size (N)	Number of SNPs	GEAS ID
Primary biliary cholangitis	NA	2015	2764	13,239	1124,241	ebi-a-GCST003129
Osteoporosis	NA	2021	3203	NA	16,380,452	finn-b-M13_OSTEOPOROSIS

GWAS = genome-wide association studies, SNPs = single nucleotide polymorphisms.

### 2.3. Estimating horizontal pleiotropy and variability

To satisfy the underlying assumptions of the selected IVs, significance levels of 5 × 10^-8^ and 1 × 10^-5^ were employed.^[16]^ The assumptions include: (i) IVs have a strong association with PBC, (ii) OP is solely influenced by IVs due to the effects of PBC, and (iii) there are no confounding variables affecting the relationship between PBC and OP through the IVs. Horizontal pleiotropy, where genetic variation influences outcomes through pathways unrelated to the exposure, can bias causal estimates in MR analysis. The MR analysis used 3 distinct analytical approaches to address this concern, each based on a different horizontal pleiotropy model. Comparing results from these methods strengthens the credibility of the findings.

### 2.4. Statistical analysis

The primary analysis employed the inverse variance weighted (IVW) method, a widely used technique in MR studies. IVW combines effect estimates from individual SNPs, weighted by their inverse variance, to assess causal effects. This method assumes that all SNPs serving as IVs are valid, meaning they have a strong association with the exposure (PBC) and are unaffected by confounding factors. If this assumption is not fully met, the random-effects IVW method is applied to correct for potential biases. To further ensure the validity of the instrumental variables, the weighted median approach is used, which requires at least 50% of the SNPs to be valid IVs. This technique accounts for variability among SNPs by assigning each estimate a weight based on its standard error, thus minimizing the impact of unreliable IVs. This approach provides reliable causation estimates even when some IVs may not be fully valid. SNP weights are used to rank them, and the median of the distribution is calculated to estimate effect sizes. If the genetic instrument is presumed to lack pleiotropic effects, MR-Egger regression is applied to determine the causal effect. MR-Egger regression assesses directional pleiotropy by examining the intercept, where a value close to 0 indicates minimal bias from pleiotropy.^[20]^ All statistical analyses were conducted using R version 4.2.2, employing the “Two Sample MR” package for MR analysis. By applying these rigorous analytical techniques, we aim to accurately estimate causal effects while addressing potential biases and variability.

## 3. Results

### 3.1. IVs selection

A total of 24 SNPs associated with PBC were identified from GWAS. Each genetic instrument related to PBC demonstrated genome-wide significance (*P* < 5 × 10^-8^) and an F-statistic above 10, indicating the absence of instrumental bias.

### 3.2. MR analysis results

**Figure 2.**
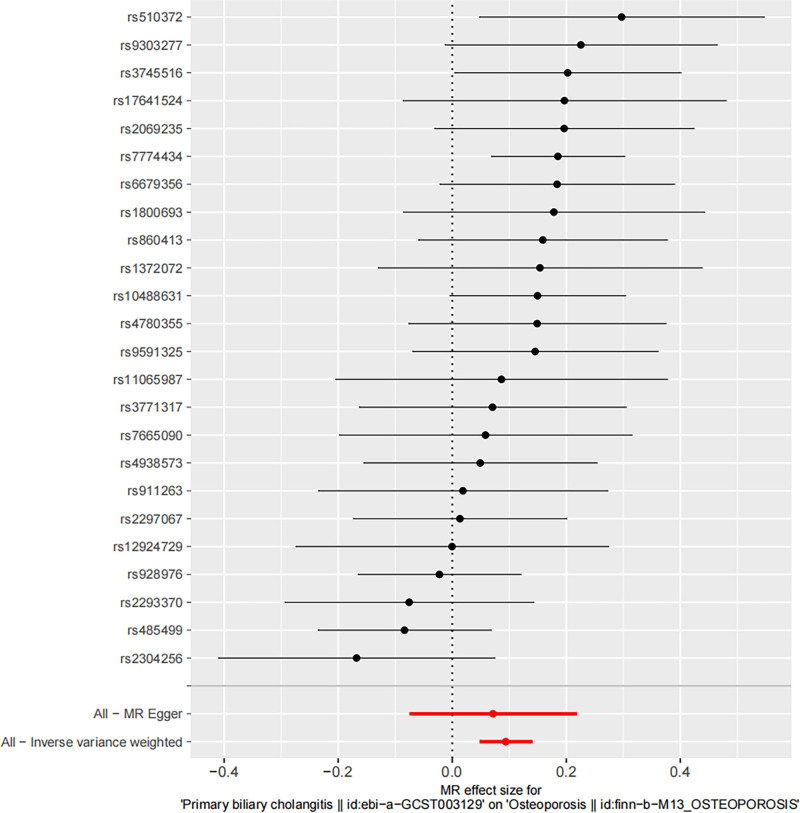
Forest plot illustrating the impact of PBC on the risk of OP. IVW = inverse variance weighted, MR = Mendelian randomization, OP = osteoporosis, PBC = primary biliary cholangitis.

**Figure 3.**
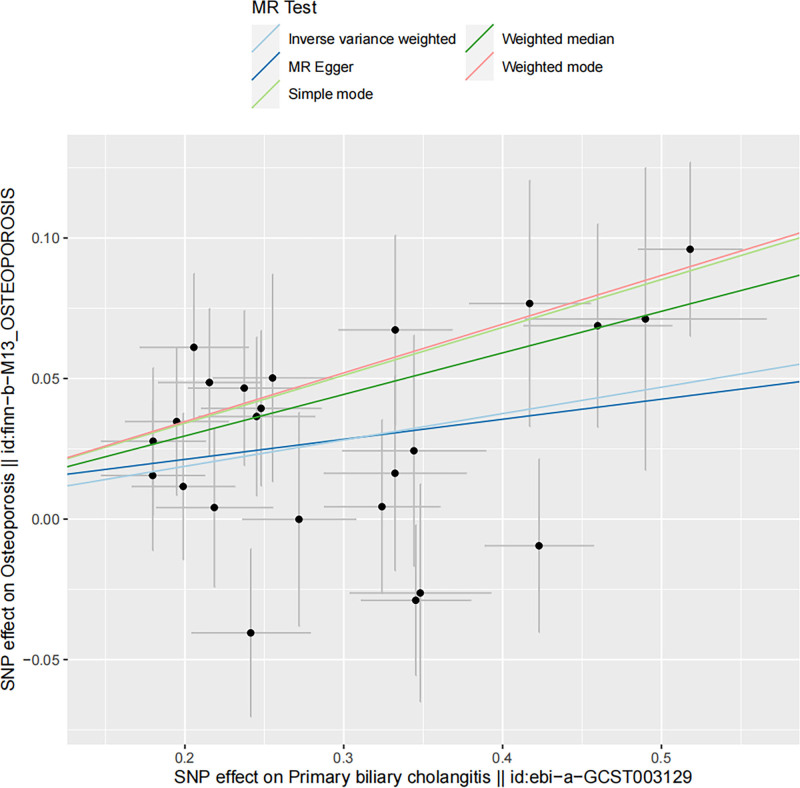
Scatter plot demonstrating the impact of PBC on the risk of developing OP. The slope of the linear line indicates the strength of the cause-and-effect relationship. IVW = inverse variance weighted, MR = Mendelian randomization, OP = osteoporosis, PBC = primary biliary cholangitis.

Using the IVW technique, a significant association was identified between PBC and OP, with an IVW odds ratio of 1.098 (95% confidence interval: 1.049–1.150) and a *P*-value of 5.41 × 10^-5^. Additional MR techniques, including MR-Egger, weighted median, weighted mode, and simple mode, also indicated associations between PBC and OP (MR-Egger OR = 1.074, 95% CI: 0.928–1.243, *P* = .348; weighted median OR = 1.159, 95% CI: 1.088–1.235, *P* = 4.31 × 10^-5^; weighted mode OR = 1.189, 95% CI: 1.047–1.351, *P* = .014; simple mode OR = 1.186, 95% CI: 1.040–1.352, *P* = .067). The findings presented in Table 2 and Figures 2 and 3 provide evidence of a direct link between PBC and OP.

**Table 2:** Mendelian randomization estimates the causal effects of PBC on OP.

Methods	nSNP	OR	95% CI	SE	*P*-value
MR-Egger	24	1.074	0.928–1.243	0.075	.348
Weighted median	24	1.159	1.088–1.235	0.032	4.31E-06
Inverse variance weighted	24	1.098	1.049–1.150	0.023	5.41E-05
Simple mode	24	1.186	1.040–1.352	0.067	.018
Weighted mode	24	1.189	1.047–1.351	0.065	.014

MR = Mendelian randomization, OP = osteoporosis, PBC = primary biliary cholangitis, SNPs = single nucleotide polymorphisms.

### 3.3. Sensitivity analysis results

**Figure 4.**
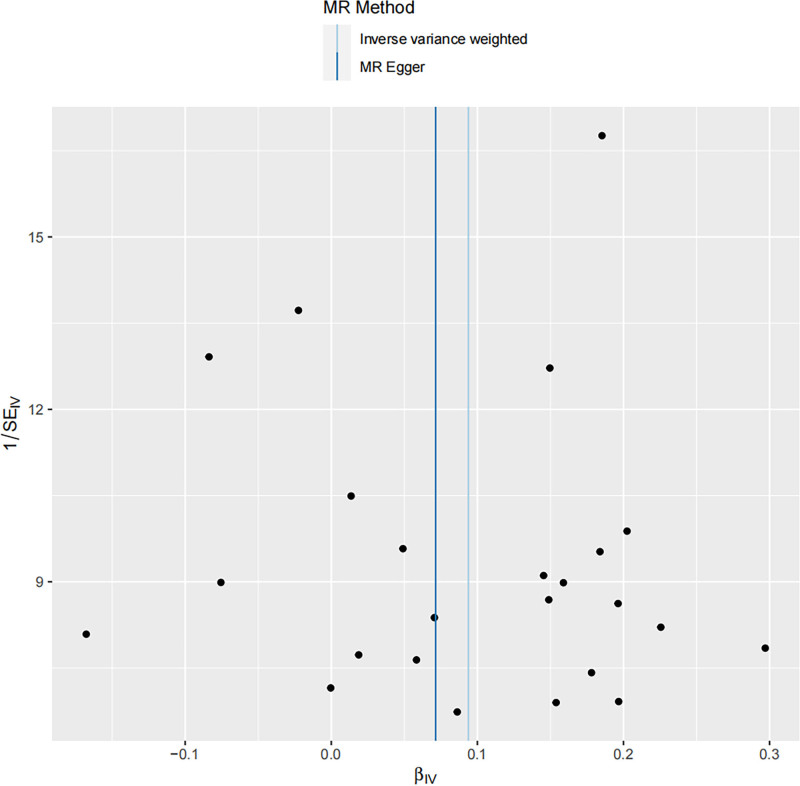
Funnel plots were used to visualize the overall heterogeneity of MR estimates regarding the effect of PBC on total OP. IVW = inverse variance weighted, MR = Mendelian randomization, OP = osteoporosis, PBC = primary biliary cholangitis.

**Figure 5.**
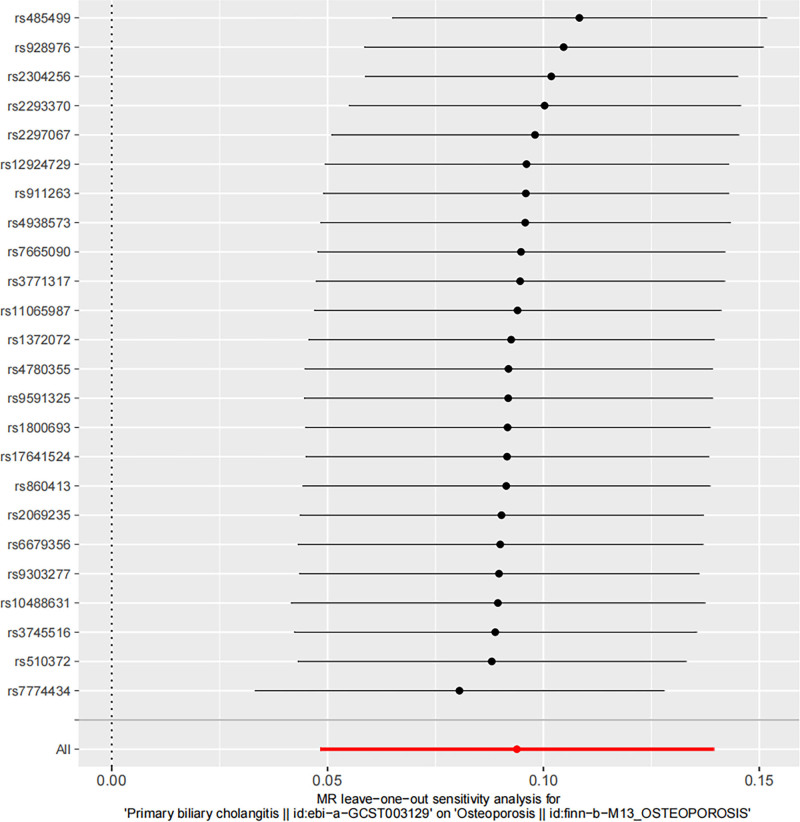
A leave-one-out plot was used to visualize the causal effect of PBC on total OP risk when excluding 1 SNP at a time. MR = Mendelian randomization, OP = osteoporosis, PBC = primary biliary cholangitis, SNPs = single nucleotide polymorphisms.

Sensitivity analysis revealed an MR-Egger intercept of 0.007 and *P*-value > .05 indicating no evidence of horizontal pleiotropy, a conclusion further supported by the funnel plot (Table 2 and Fig. 4). In addition, MR heterogeneity assessments showed no significant differences among SNPs (MR-Egger Q = 27.369, *P* = .198; IVW Q = 27.494, *P* = .236). The stability of the results was validated through leave-one-out analysis, demonstrating robustness and minimal influence from individual SNPs (Fig. 5).

## 4. Discussion

The high prevalence of OP in individuals with PBC significantly exceeds that observed in the general population, indicating a strong association between the 2 conditions. Research has demonstrated that OP is a notable complication among PBC patients, especially in postmenopausal women.^[21]^

The relationship between PBC and OP is not well understood and may vary depending on the severity of the disease. Recent research indicates that individuals with PBC face a significantly higher risk of developing OP, with a relative risk of 2.79 when compared to healthy individuals.^[22]^ This increased risk is believed to be associated with several factors, particularly the autoimmune characteristics of PBC, which may disrupt normal bone metabolism. Additionally, there is documented evidence linking liver diseases, such as cirrhosis, to OP, suggesting that liver dysfunction can negatively impact bone health.^[23]^ In patients suffering from liver cirrhosis, the occurrence of OP is considerably more frequent, highlighting the importance of liver disease severity in OP development.^[23]^ Furthermore, vitamin D deficiency is prevalent among patients with PBC and may further heighten the risk of OP.^[13]^ The complex interactions between vitamin D levels, bone mineral density (BMD), and the severity of liver disease illustrate the challenges in managing OP in individuals with PBC.

This research demonstrates that PBC increases the risk of developing OP, supported by genetic data that highlights the influence of PBC on an individual’s susceptibility to OP. The consistency of results obtained through various statistical methods enhances the credibility of these findings.

PBC significantly increases low bone mass and OP prevalence, raising fracture rates; studies show 20% to 44% of PBC patients have OP, with higher rates in advanced disease. A European cohort study found PBC patients face greater fracture risks and mortality than matched controls, while a Chinese study reported 45% OP prevalence, linking it to older age, low body mass index, and advanced disease.^[21,24]^ Clinical guidelines recommend vigilant bone health monitoring, including a baseline DXA scan at PBC diagnosis and reassessments every 2 years, especially for those with additional risk factors. Early detection of bone density loss enables timely preventive and therapeutic measures to lower fracture risk.

Management of OP in PBC follows standard care, emphasizing lifestyle counseling, nutritional support, and routine supplementation. Patients should engage in weight-bearing exercises, avoid smoking and excessive alcohol, and ensure adequate calcium and vitamin D intake while monitoring fat-soluble vitamins. Supplementation of calcium (1200–1500 mg) and vitamin D (800–1000 IU) is recommended, with checks on vitamin D levels. Pharmacologic therapy is necessary for those with OP or fragility fractures. Bisphosphonates are first-line for PBC-related OP, improving bone density, but caution is needed with oral forms in patients with esophageal varices; intravenous options may be better. Hormone replacement therapy shows benefits but is rarely used due to risks. Calcitriol has been tested but is ineffective for fractures or BMD improvement. PBC OP differs from postmenopausal types, driven by low bone formation, limiting antiresorptive treatments. Targeted therapies are needed, and proactive management is crucial for better outcomes. Future research may lead to specialized treatments.^[25,26]^

The relationship between PBC and OP is complex, involving various factors such as hormonal imbalances, liver dysfunction, changes in the gut microbiome, and reduced nutrient absorption. Hormonal imbalances, especially those linked to estrogen, are crucial for maintaining bone health. In postmenopausal women, lower estrogen levels can result in increased bone resorption and decreased bone formation, which significantly contributes to the development of OP.^[27]^ Additionally, liver dysfunction related to PBC can interfere with the metabolism of essential hormones for bone density, including vitamin D and estrogen.^[28]^ The liver plays a vital role in converting vitamin D into its active form, and any decline in liver function can lead to deficiencies that adversely affect bone health.^[29]^

Changes in the gut microbiome may play a significant role in this context. Dysbiosis, which refers to an imbalance in gut microbiota, has been associated with several metabolic disorders, including OP. The gut microbiota significantly affects the absorption of vital nutrients necessary for maintaining bone health, such as calcium and vitamin D.^[30]^ In patients with PBC, shifts in the composition of gut microbiota can hinder nutrient absorption, thereby increasing the risk of developing OP.^[31]^

The gut–liver axis is crucial in understanding the relationship between liver health and bone metabolism. The gut microbiota can influence liver function, and in turn, liver health can affect the gut, creating a feedback loop that impacts both systems.^[29]^ For example, specific metabolites produced by gut bacteria can alter liver inflammation and fibrosis, conditions often seen in PBC. This interaction can significantly affect overall metabolic health and the integrity of bones.^[32]^

The interaction between osteopontin and the receptor activator of nuclear factor κB ligand (RANKL)-receptor activator of nuclear factor κB pathway plays a critical role in maintaining bone metabolism through the balanced activities of osteoclasts and osteoblasts. Osteopontin plays a crucial role in regulating osteoclast activity, which in turn affects the dynamics of bone resorption. In the context of PBC, the liver’s diminished production of osteoprotegerin (OPG) disrupts the delicate balance between bone formation and resorption. OPG is vital as it inhibits osteoclast differentiation and activity; when OPG levels are low, osteoclast activity becomes unchecked, leading to increased bone degradation. This situation is particularly alarming as it contributes to the development of OP, a condition marked by reduced bone quality and a heightened risk of fractures.^[26]^ The pathophysiology of bone loss in PBC is complex, involving not just the direct impact of reduced OPG but also the interactions between inflammatory processes and altered signaling pathways. For example, chronic liver disease can elevate levels of RANKL, which further promotes osteoclastogenesis and bone resorption.^[33]^ The disruption of the OPG/RANKL system is a recurring theme in various osteolytic conditions, underscoring the necessity of maintaining this balance for optimal bone health.^[34]^ Additionally, the systemic effects of liver dysfunction extend beyond OPG levels; the gut–bone axis has also been implicated in bone metabolism. The gut microbiome and its metabolites can significantly influence bone health, and changes in this axis may worsen bone loss in individuals with liver diseases.^[7]^ Therefore, it is crucial to understand how liver dysfunction impacts bone metabolism to develop targeted therapeutic strategies aimed at reducing bone loss in patients with PBC and other chronic liver diseases.^[26]^

Some scientists propose that OP linked to PBC may mainly arise from hindered bone formation. In patients with PBC, elevated bilirubin levels and increased bile acids could interfere with the production of bone morphogenetic proteins, which play a crucial role in bone development and regeneration.^[11]^ This interference might activate apoptosis pathways, reduce the activity of osteogenic transcription factors, and impede bone formation, all of which could lead to decreased bone density in those affected.^[24]^

In PBC, cholestasis significantly hinders the absorption of fat-soluble vitamins, particularly vitamins A, D, E, and K.^[35]^ These vitamins are vital for various physiological functions, including the maintenance of bone health. For example, vitamin D is essential for calcium absorption and bone mineralization; a deficiency in this vitamin not only disrupts calcium balance but also worsens liver inflammation and fibrosis.^[35]^ Vitamin K plays a very important role in bone metabolism and the regulation of calcium within the bones.^[36]^ A deficiency in vitamin K can impede the activation of bone-forming proteins like osteocalcin, which ultimately weakens bone strength and stability, thereby increasing the risk of OP in patients with PBC.^[37]^ Additionally, vitamin A supports bone health by influencing the activity of osteoblasts and osteoclasts, the cells responsible for bone formation and resorption.^[38]^ Vitamin E, on the other hand, serves as an antioxidant, shielding bone cells from oxidative stress that can contribute to bone loss.^[39]^ Consequently, patients with cholestasis, including those with PBC, face an elevated risk of deficiencies in these vitamins, which can worsen bone health and heighten the likelihood of OP and fractures.^[40]^ Therefore, it is important to monitor and supplement fat-soluble vitamins in individuals with cholestasis to mitigate these risks and promote overall bone health.^[41]^

Intestinal microbiota alterations significantly contribute to the development of both PBC and OP. Disruptions in the gut microbiota, especially during the uncompensated phase of PBC cirrhosis, can initiate inflammatory responses and activate the immune system. These changes disrupt hormone regulation and nutrient absorption, affecting the intestinal–hepatic–bone axis. Consequently, bone metabolism may be compromised, leading to an increased risk of OP in individuals with PBC.^[42]^ Recent studies have underscored the complex relationship between gut microbiota and various diseases, including PBC, where dysbiosis may play a role in immune-mediated liver damage. Patients with PBC show significant alterations in gut microbiota composition, which can facilitate the translocation of pathogenic bacteria and their metabolites, ultimately resulting in abnormal autoimmune activation and liver injury.^[43]^

OP is marked by a reduction in BMD and a heightened risk of fractures. Recent studies indicate a two-way relationship between gut microbiota and bone health. The gut microbiota can impact bone metabolism through the production of microbial metabolites, including short-chain fatty acids, which play a role in bone remodeling processes.^[44]^ Additionally, the deficiency of estrogen following menopause has been associated with an imbalance in gut microbiota, potentially worsening bone loss and playing a role in the development of OP.^[45]^

The interplay between gut microbiota and the endocrine system plays a significant role in both PBC and OP. In the case of PBC, changes in gut microbiota can affect liver function and immune responses, potentially exacerbating the condition. Similarly, in OP, the gut microbiota influences hormonal regulation and inflammation, which are critical factors for maintaining bone health.^[43,45]^ By understanding these intricate relationships, researchers can explore new therapeutic strategies that target gut microbiota, offering promising approaches for the effective management of both PBC and OP.

Liver dysfunction, especially hepatic fibrosis associated with PBC, significantly impacts bone health by reducing bone density and elevating the risk of OP. This condition diminishes the liver’s ability to synthesize essential substances, which adversely affects the metabolism of crucial hormones that regulate bone health, including vitamin D. When the liver’s capacity to produce vitamin D is compromised, it leads to inadequate calcium absorption and interferes with the process of bone mineralization. As a result, these disruptions contribute to lower bone density and a heightened risk of developing OP.^[46]^ Studies indicate that patients with PBC, even when cirrhosis is not present, show a greater prevalence of OP compared to healthy individuals. For example, a case–control study revealed that postmenopausal women diagnosed with PBC had a significantly higher rate of OP than their matched controls, underscoring the detrimental effects of liver disease on bone health.^[21]^

The mechanisms behind this association may involve an imbalance in bone turnover. This is supported by findings that show elevated levels of bone-specific alkaline phosphatase in patients with PBC, which indicates increased bone formation. However, the markers for bone resorption exhibited variability.^[21]^ Furthermore, advanced fibrosis in PBC has been associated with low bone turnover, suggesting a correlation between the severity of liver disease and the deterioration of bone health.^[21]^

The relationship between liver fibrosis and BMD has been clarified in studies examining nonalcoholic fatty liver disease, revealing that significant liver fibrosis is independently linked to lower BMD.^[47]^ This indicates that hepatic fibrosis, irrespective of the specific liver condition, may be a crucial factor in the development of OP. In conclusion, hepatic fibrosis in PBC and other liver diseases significantly contributes to reduced bone density and an elevated risk of OP, highlighting the need for vigilant monitoring and management of bone health in these patients.^[48]^

Our research underscores the increased risk of OP in individuals with PBC, a finding that is strongly supported by genetic evidence. This highlights the importance of early diagnosis and effective management of OP in patients with PBC.^[49]^ Through MR studies, we have clarified the relationship between PBC and OP. However, it is important to acknowledge the limitations of MR studies. Firstly, the GWAS data used in this study were obtained from European populations, and the findings may not be fully applicable to East Asian populations, American populations, and other ethnic groups. Secondly, MR analysis may not completely eliminate confounding factors, particularly those associated with exposure or outcome variables. Moreover, the effectiveness of MR analysis can be affected by the strength of the instrumental variables, with weak genetic variants potentially resulting in inadequate power. Thirdly, the small number of SNPs (N = 24) may lead to decreased statistical power and diminished capability to detect heterogeneity. These factors emphasize the necessity for larger, more inclusive studies that encompass a variety of populations to enhance our understanding of the underlying mechanisms linking PBC and OP. Despite these challenges, our research provides significant insights into the connection between PBC and OP, providing a theoretical basis for clinicians to develop appropriate clinical interventions.

## 5. Conclusion

In conclusion, this study conducted two-sample MR study to identify the potential causal relationships between OP and PBC. The findings indicated that the level of PBC was positively correlated with OP, and more research will be necessary to confirm this correlation in the future.

## Acknowledgments

The authors would like to thank all the genetics alliances for generously sharing the GWAS summary data with the public.

## Author contributions

**Data curation:** Weinan Liu, Jian Liu, Jiazhong Lin, Shoubao Wang.

**Writing – original draft:** Shoubao Wang.

**Writing – review & editing:** Weinan Liu, Yanling Dai.
